# Hysteroscopic management of complete septate uterus with septate cervix, and longitudinal vaginal septum: a case report

**DOI:** 10.1093/jscr/rjae238

**Published:** 2024-04-18

**Authors:** Nahida Hama Ameen Ahmed, Nasren Sharef Sabr, Rawa Bapir, Dilan S Hiwa, Ali H Hasan, Rawezh Q Salih, Soran H Tahir, Berun A Abdalla, Snur Othman, Fahmi H Kakamad

**Affiliations:** Smart Health Tower, Madam Mitterrand, Sulaymaniyah, Kurdistan 46001, Iraq; Sulaymaniyah Maternity Teaching Hospital, Sulaymaniyah, Kurdistan 46001, Iraq; Smart Health Tower, Madam Mitterrand, Sulaymaniyah, Kurdistan 46001, Iraq; Smart Health Tower, Madam Mitterrand, Sulaymaniyah, Kurdistan 46001, Iraq; Department of Urology, Sulaymaniyah Surgical Teaching Hospital, Sulaymaniyah, Kurdistan 46001, Iraq; Kscien Organization for Scientific Research (Middle East office), Hamid Street, Azadi Mall, Sulaymaniyah, Kurdistan 46001, Iraq; Smart Health Tower, Madam Mitterrand, Sulaymaniyah, Kurdistan 46001, Iraq; Smart Health Tower, Madam Mitterrand, Sulaymaniyah, Kurdistan 46001, Iraq; Sulaymaniyah Directorate of Health, Sulaymaniyah, Kurdistan 46001, Iraq; Smart Health Tower, Madam Mitterrand, Sulaymaniyah, Kurdistan 46001, Iraq; Kscien Organization for Scientific Research (Middle East office), Hamid Street, Azadi Mall, Sulaymaniyah, Kurdistan 46001, Iraq; Smart Health Tower, Madam Mitterrand, Sulaymaniyah, Kurdistan 46001, Iraq; College of Medicine, University of Sulaimani, Madam Mitterrand Street, Sulaymaniyah, Kurdistan 46001, Iraq; Smart Health Tower, Madam Mitterrand, Sulaymaniyah, Kurdistan 46001, Iraq; Kscien Organization for Scientific Research (Middle East office), Hamid Street, Azadi Mall, Sulaymaniyah, Kurdistan 46001, Iraq; Kscien Organization for Scientific Research (Middle East office), Hamid Street, Azadi Mall, Sulaymaniyah, Kurdistan 46001, Iraq; Smart Health Tower, Madam Mitterrand, Sulaymaniyah, Kurdistan 46001, Iraq; Kscien Organization for Scientific Research (Middle East office), Hamid Street, Azadi Mall, Sulaymaniyah, Kurdistan 46001, Iraq; College of Medicine, University of Sulaimani, Madam Mitterrand Street, Sulaymaniyah, Kurdistan 46001, Iraq

**Keywords:** complete septate uterus, cervical septum, longitudinal vaginal septum, hysteroscopic management

## Abstract

Mullerian anomalies occur as a result of errors during embryogenesis. The estimated incidence of these anomalies is around 1% in the general population and 3% in women complaining of suboptimal reproductive outcomes and infertility. A 21-year-old female patient was referred to our hospital due to primary infertility for 18 months. After a proper history, physical examination and further diagnostic steps, including ultrasound and magnetic resonance imaging, a diagnosis of complete septate uterus with septate cervix and longitudinal vaginal septum was made. Following hysteroscopic resection of all the septa and two cycles of ovulation induction, the patient was able to conceive. However, she needed cervical cerclage later due to cervical insufficiency. The baby was delivered at term and was healthy. A uterine, cervical and longitudinal vaginal septum is a unique entity of Mullerian anomalies. Resection of all septa through a hysteroscopic approach resulted in a good outcome for our patient.

## Introduction

Congenital anomalies in the female reproductive system result from embryogenic errors, occurring in about 1% of the general population and 3% of women with fertility issues [1]. The most common anomaly is uterine, with an estimated incidence of 5–25% in recurrent pregnancy loss and 4% in the general population [2]. Approximately 60–80% of septate uterus cases lead to spontaneous abortion, with a fetal survival rate of 6–28% [3–7]. Management involves hysteroscopic septoplasty, but controversy surrounds which septa to resect and the actual benefit of the intervention. We present a unique case of complete septate uterus, septate cervix and longitudinal vaginal septum, treated with hysteroscopic complete septa resection for primary infertility.

## Case presentation

### Patient information

A 21-year-old female patient was referred to our hospital due to primary infertility for 18 months. The patient’s menarche was at the age of 14, with regular menses every 30 days, lasting for 5 days. However, the patient also had complaints of dysmenorrhea and dyspareunia. She has a history of hypothyroidism, which was well controlled by treatment. No other significant past medical or surgical history was noted. It is worth mentioning that the patient’s family history was negative for any known anomaly.

### Clinical finding

Upon physical examination, a complete longitudinal vaginal septum was detected. Otherwise, no abnormality was noted.

### Diagnostic approach

**Figure 1:**
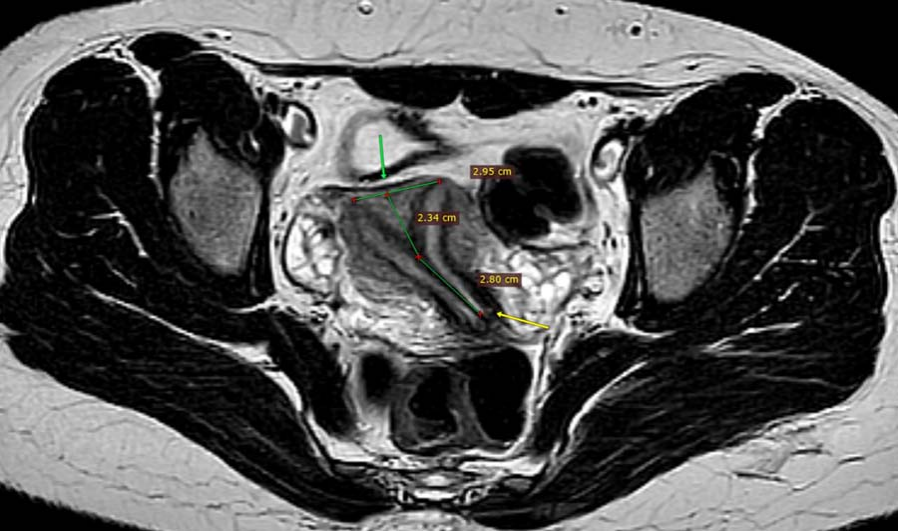
MRI pelvis, axial T2, complete separate uterine cavity (complete septate uterus), two cervices openings (below arrow), no fundal cleft, inter-cornual distance = 29.5 mm (above arrow), the upper 23.5 mm of the septum is composed of myometrium, while the distal 28 mm is composed of fibrous tissue.

The patient underwent a comprehensive diagnostic workup. A hormonal assay of the patient showed that follicle-stimulating hormone, luteinizing hormone and thyroid-stimulating hormone were all within the normal range, except for prolactin level, which was elevated (64.72 ng/ml reference range 4.79–23.3). In addition, pelvic and transvaginal ultrasounds were performed, but they were inconclusive. Pelvic magnetic resonance imaging (MRI) revealed a uterus that was normal in size, with two uterine cavities separated by a septum of low T2 signal, extending into the endocervical canal (Fig. 1). The surface of the fundus was convex in shape with an acute angle between uterine cavities, which was about 60°. A thin regular endometrium about 7 mm in each cavity was seen. Hysteroscopy showed a complete vaginal septum, cervical septum and a septum inside the uterus, reaching the fundus and separating the uterus into two separate cavities. A normal outer wall of the uterus was observed during laparoscopy of the patient.

### Therapeutic intervention

The complete septum inside the uterus, cervical canal, and the longitudinal vaginal septum were removed with cauterization through a hysteroscopic approach. An intrauterine contraceptive device was inserted, and she was put on contraceptive pills for 3 months. After 3 months, another hysteroscopy was performed, and the remnants of the septa were removed. She was advised to try to conceive naturally. However, after failed attempts at conceiving naturally, two trials of ovulation induction were performed, and she became pregnant afterward.

### Follow-up and outcome

Three months into the pregnancy, Shirodkar cervical cerclage was performed due to cervical insufficiency caused by the previous cauterization. The patient was kept under close supervision throughout the pregnancy and the baby was delivered at term and both the baby and the mother are healthy now. The baby was born without any complication and both the mother and the baby are healthy now.

## Discussion

The widely accepted hypothesis for Mullerian development involves a complex multistep pattern from the 11th to 13th week of intrauterine life. Most uterine anomalies are associated with a single cervix, challenging traditional theories. Mangla et al. and Duffy et al. present cases challenging consensus on embryological patterns [8, 9].

Mullerian anomalies can be asymptomatic or present with various symptoms, including trouble conceiving. Diagnosis, though not classified by the American Fertility Society, calls for formal recognition due to recent literature describing similar cases. Misdiagnoses, such as uterine didelphys resemblance, highlight the need for MRI, the standard imaging test for uterine abnormalities. Concurrent urinary system anomalies, occurring in up to 30% of cases, should be assessed for comprehensive management [10–12].

Management of complete uterine, cervical and longitudinal vaginal septum lacks consensus and has limited data. Chen et al.’s review showed varied success with uterine septum resection, while Duffy et al. achieved success with vaginal septum resection to enhance cervix access. Praveen et al.’s approach of resecting uterine and vaginal septa alone was unsuccessful [9, 13, 14]. We opted for complete hysteroscopic septa resection, addressing dyspareunia and the association with infertility. Our patient conceived after two ovulation induction cycles but needed Shirodkar cervical cerclage due to cervical insufficiency from hysteroscopy.

## Conclusion

A complete uterine, cervical and longitudinal vaginal septum is a rare entity of Mullerian anomalies. Complete resection of all the septa through a hysteroscopic approach resulted in a good outcome for our patient. However, more research is needed to determine this entity’s most optimal treatment plan.
